# Transcriptome Analysis of Ovary Development in Nile Tilapia Under Different Photoperiod Regimes

**DOI:** 10.3389/fgene.2019.00894

**Published:** 2019-09-19

**Authors:** Zhanyang Tang, Yi Zhou, Jun Xiao, Huan Zhong, Weiwei Miao, Zhongbao Guo, Xu Zhang, Lei Zhou, Yongju Luo

**Affiliations:** ^1^State Key Laboratory for Conservation and Utilization of Subtropical Agro-bioresources, College of Animal Science and Technology, Guangxi University, Nanning, China; ^2^Guangxi Academy of Fishery Sciences, Nanning, ChinaEdited by: Enrique Medina-Acosta, Universidade Estadual do Norte Fluminense Darcy Ribeiro, Brazil

**Keywords:** Nile tilapia, gonad development, photoperiod, transcriptome, high-throughput sequencing

## Abstract

This study investigated the molecular mechanisms involved in ovarian transcriptomic responses in Nile tilapia under different photoperiod regimes. Histological analysis indicated that ovarian development was significantly affected by photoperiod. The photoperiods tested were as follows: LD (12 h light:12 h dark), LL (24 h light:0 h dark), and DD (0 h light:24 h dark). The longer photoperiod (LL) was shown to induce ovary development earlier than LD and DD. Next, ovary transcriptome levels were sequenced and analyzed. These data indicated that 988, 992, and 1,036 differentially expressed genes (DEGs) were detected by comparing LD–LL, LD–DD, and LL–DD. A number of genes that may be involved in photoperiod-specific regulation of ovarian development were observed. These findings may be useful for investigating the molecular mechanisms underlying light-induced ovarian development in Nile tilapia.

## Introduction

Nile tilapia is one of the most important commercial fish worldwide and is prized as an aquaculture species (Biswas et al., 2005; Fernandes et al., 2015). In aquaculture systems, ovary development is crucial in producing sexually mature females for fish reproduction. It has been established in the literature that light regulates ovary development *via* the brain–pituitary–gonad (BPG) axis. Specifically, ovary development in Nile tilapia has been shown to be controlled by rhythmic signals induced by light (Biswas et al., 2005; Rad et al., 2006; Veras et al., 2013).

Individual female tilapia exhibit unique ovarian developmental patterns; thus, they do not reproduce synchronously, i.e., the spawning cycle is not uniform across individuals (Bhujel, 2000; Coward and Bromage, 2000; Little and Hulata, 2000). In order to increase tilapia seed production and obtain a homogeneous stock of fry, the hatchery typically increases the number of broodfish. Although the use of high numbers of broodfish helps overcome these problems, this method increases the costs needed to house and maintain fish. In addition, manipulating the photoperiod has been successful in altering the tilapia reproductive cycle (Bromage et al., 2001). In the case of tilapia and other tropical fish species (Yumnamcha et al., 2017; Khan et al., 2018), manipulating the photoperiod, including varying the light intensity, can have a positive effection reproduction. Ridha et al. (1998) reported that longer and brighter days (18 days) resulted in more fry and improved spawning synchrony in Nile tilapia compared with shorter days and lower light intensity. Campos-Mendoza (Campos-Mendoza et al., 2004) showed that longer day lengths help to alleviate the production problems caused by low fecundity and poor spawning synchrony and improves reproductivity in Nile tilapia. However, the precise molecular mechanism of light-induced ovarian development in female tilapia has not been elucidated.

High-throughput sequencing methods offer whole genome expression proﬁling, genome annotation, and discovery of non-coding RNA (Mutz et al., 2013). With the emergence of high-throughput (next-generation) sequencing, RNA sequencing (RNA-Seq) can be applied to transcriptome studies across the whole genome. The differences in gene expression patterns across tissue types and environmental conditions can be observed using transcriptome analysis. For example, gene expression generally changes under abiotic stress, especially during disease and environmental challenges in aquatic animals (Mininni et al., 2014; Carmona et al., 2016). Therefore, RNA-Seq has been widely used to investigate differential gene expression and molecular pathways under certain environmental stressors (Dugas et al., 2011; Teets et al., 2012). Transcriptome profiling at the whole genome expression level is an efficient way to evaluate biological response processes under different environmental conditions in Nile tilapia (Sun et al., 2014; Xu et al., 2015).

In this study, high-throughput data for responses in female Nile tilapia under different photoperiod regimes were obtained using an Illumina sequencing 2500 platform. Transcriptome sequences from ovaries were mapped to a reference genome. Functional annotation and gene ontology (GO) analyses were performed, and candidate genes involved in the responses were identified. These results provide a valuable resource for understanding the mechanisms of light-induced responses in female Nile tilapia. These data may also serve in facilitating genetic improvement of ovarian development in commercially important species of fish.

## Materials and Methods

### Animals and Treatment

Twenty two-year-old Nile tilapia (15 females and 5 males) were collected from the Guangxi Academy of Fishery Sciences (Nanning, China) and reared in a 50-m^3^ pond. After 1 week, 20 tilapia were divided into 5 groups. Each group contained three females and one male cultured in a net cage (2-m length × 2-m width) under a natural photoperiod and water temperature (25–28°C). The mouths of female fish were checked every 5 days for brooded embryos. Any embryos in the mouth were removed and cultivated artificially in an incubator; each family’s brood was cultivated in a separate incubator. There was one aerating stone in each incubator, and the water temperature was controlled at 26–28°C. Five families were obtained in the reproductive phase. One family fry was randomly selected for subsequent experiments. These fry were cultured in a 180-m^3^ pond for 50 days. Then, females were randomly selected for following study with the reproductive phase as II period detected by histological analysis.

Thirty healthy female tilapia (post-hatch age of 50 days and mean weight of 30 g) were randomly assigned to three groups. Group 1 was exposed to 12 h light:12 h dark (LD), group 2 was exposed to 24 h light:0 h dark (LL), and group 3 was exposed to 0 h light:24 h dark (DD). Group 1 served as the control, while group 2 and group 3 served as the treatment groups. Before the experiment, 30 fish were cultured for 1 week under a photoperiod of 12 h light:12 h dark. Fish were maintained in aerated plastic buckets (volume of 50 L) for 30 days and were fed twice daily. Plastic buckets were placed in a dark room equipped with a safelight lamp (OPPLE, 220V, 3W). The safelight lamp was turned on when feeding and sampling. The water temperature was maintained at 26–28°C in plastic buckets throughout the study. The plastic buckets were covered with a black cloth throughout the study. The only light source was an LED lamp (OPPLE, 220V, 12W) with a light intensity of 1,000 lx. The LED light was controlled by a timer switch in group 1 (12 h light:12 h dark). The LED was illuminated throughout the day in group 2 (24 h light:0 h dark).

### Tissue Sampling and RNA Extraction

After the experiment, fish were anesthetized with MS-222 and then sacriﬁced. Ovary sampling of group 1 (12 h light:12 h dark) and group 2 (24 h light:0 h dark) was carried out under an LED lamp (OPPLE, 220V, 12W). Ovary sampling of group 3 (0 h light:24 h dark) was carried out under safelight lamp (OPPLE, 220V, 3W) to preserve the gene expression level. Three ovaries were randomly selected in each trial group and immersed in Bouin’s solution for histological analysis. In the remaining test fish, two ovaries were randomly selected in each trial group and frozen in liquid nitrogen for RNA extraction. Total RNA was extracted using a TRIzol Kit (Invitrogen, Carlsbad, CA, USA) following the manufacturer’s instructions. RNA samples were digested by DNase I to remove possible genomic DNA contamination for cDNA synthesis and sequencing.

### Histological Analysis

Histological differences in ovaries from photoperiod-treated and control fish were investigated. The fixed ovarian samples were dehydrated and embedded in paraffin, 5-µm-thick (paraffin) sections were cut, and the paraffin sections were stained with hematoxylin–eosin (HE) for histological examination with a light microscope (Sun et al., 2016).

### cDNA Library Construction and Sequencing

Sequencing libraries were generated using a NEBNext Ultra*™* RNA Library Prep Kit for Illumina (New England Biolabs, Ipswich, MA, USA) following the manufacturer’s recommendations. Index codes were added to attribute sequences to each sample. In order to select cDNA fragments of constructs preferentially 200–250 bp in length, the library fragments were purified with the AMPure XP system (Beckman Coulter, Beverly, MA, USA). PCR products were also purified (AMPure XP system). Library quality was evaluated using the Agilent Bioanalyzer 2100 system (Agilent, Santa Clara, CA, USA). Subsequently, the cDNA library was sequenced on an Illumina HiSeq 2500 platform after the test paired-end reads were generated.

### Read Mapping and Differential Expression Analysis

Raw data (raw reads) of the FASTQ format were first processed through in-house Perl scripts. In this step, clean data (clean reads) were obtained by removing reads containing adapters, reads containing ploy-N (the proportion of N > 10%), and low-quality reads (the proportion of basic group with mass value Q ≤ 10 > 50% in the whole reads) from the raw data. All downstream analyses were based on clean data with high quality. The reads were processed and aligned with the *Oreochromis niloticus* reference genome (ftp://ftp.ncbi.nlm.nih.gov/genomes/Oreochromis_niloticus/Assembled_chromosomes/seq/) using TopHat2 software (http://ccb.jhu.edu/software/tophat/index.shtml) (Kim et al., 2013). TopHat2 initially removed a portion of reads based on the quality of information accompanying each read and then mapped the reads to the reference genome. Differential expression analysis of two groups was performed using the DESeq package (1.10.1) (http://www.bioconductor.org/packages/release/bioc/html/DESeq.ht) (Anders and Huber, 2010). DESeq provides statistical routines for determining differential expression in digital gene expression data using a model based on the negative binomial distribution. Gene expression levels were estimated using FPKM values (fragments per kilobase of exon per million fragments mapped) using the Cufflinks (http://cufflinks.cbcb.umd.edu/) software. The resulting P-values were adjusted using the Benjamini–Hochberg’s approach for controlling the false discovery rate. Genes with an adjusted P-value < 0.01 and fold change of FPKM value ≥ 2 found by DESeq were considered differentially expressed.

### Gene Functional Annotation

Gene function was annotated based on the following databases: Nr (NCBI non-redundant protein sequences), Nt (NCBI non-redundant nucleotide sequences), KO (KEGG Ortholog database), and GO (Gene Ontology). GO enrichment analysis of the differentially expressed genes (DEGs) was implemented using the GOseq R packages based on the Wallenius non-central hyper-geometric distribution (Young et al., 2010), which can adjust for gene length bias in DEGs. Kyoto Encyclopedia of Genes and Genomes (KEGG) is a database resource for unveiling pathways in genes (Kanehisa et al., 2007). We used KOBAS (http://kobas.cbi.pku.edu.cn/) (Mao et al., 2005) software to test the statistical enrichment of DEGs in KEGG pathways.

### Quantitative Real-Time PCR Analysis

Total RNA was extracted using an EZgene™ Biozol RNA Kit (Biomiga, USA) from nine ovary samples (n = 3 for LL, LD, and DD groups each) and analyzed by agarose gel and spectrophotometer. cDNA was synthesized from 20 μl of total DNA-free RNA using the First Strand cDNA Synthesis Kit (Biomiga, USA) using 1 μg of total RNA. Primers for selected genes were designed by primer 5.0 (**Table 1**). Quantitative real-time PCR (qRT-PCR) was carried out using the ABI 7500 qRT-PCR System (ABI, USA). The qRT-PCR detection system was performed using a QuantiNova™ SYBR^®^ Green PCR Kit (Qiagen, Hilden, Germany), which was incubated at 95°C for 2 min, followed by 40 cycles of 95°C for 5 s, and 60°C for 30 s. For qRT-PCR experiments, three biological replicates and three technical replicates were performed for each sample. β-Actin was used as the reference gene to determine the relative expression. Gene expression levels were analyzed based on the 2^−ΔΔCt^ method.

**Table 1:** Test of primer sequence of genes validated by qRT-PCR in Nile Tilapia *(Oreochromis niloticus)*.

List	Name	Gene sequence (5’ to 3’)
1	fshbF	TCGACACCACCATTTGTGAA
2	fshbR	TATTTCACCTCGTAGGACCACTCTC
3	gadd45g-F	CAGGACTACCTGACAGTTGGAGTTT
4	gadd45g-R	AACGCCTGGATGAGAGTGAAG
5	pgr-F	TGATGCTTGGAGGGAGGAA
6	pgr-R	TCATTTGTTGGGAGAGTTGTAGG
7	cacna1dF	CAAAGTCCAGCCCAGATGAGT
8	cacna1dR	CGATGCGGTAGTTGTAGATAGGG
9	adcy8F	GTTCCTCCTCGCCGTGTTT
10	adcy8R	GTGTTCTCGCAGTTCCTTCATCT
11	p53-F	CGGCACCAAAGAGATGAAAA
12	p53-R	CCACGAACAGGGAGGAGAA
13	β-actin-F	CCACAGCCGAGAGGGAAAT
14	β-actin-R	CCATCTCCTGCTCGAAGTC

## Results

### Illumina Sequencing Data and Mapping

To obtain a comprehensive understanding of the difference in light-induced expression in the three groups of female Nile tilapia, we sequenced RNA samples from fish ovaries. After filtering low-quality reads from the raw reads, 44,932,587 clean reads (LD), 23,635,959 clean reads (LL), and 22,735,011 clean reads (DD) were obtained. There was a similar Q30 percentage (nucleotides with a quality value larger than 30) over 88% and a GC percentage over 48% (**Table 2**). These clean reads were further assembled into unigenes and aligned with the reference genome. There were approximately 89.8, 47.2, and 45.4 million total reads generated for each sample (**Table 3**). The results showed that the percentage of mapped reads to reference genomes was 75.94, 75.18, and 77.70% in LD, LL, and DD RNA samples, respectively. These results indicated that the samples adequately represented all of the transcripts under the present experimental conditions. All of the RNA-Seq data generated in this study have been deposited in the NCBI SRA database (SRA accession: SRP150481).

**Table 2:** Sequencing data statistics.

Samples	Clean reads	Clean bases	GC content	%≥Q30
LD	44,932,587	13,029,966,264	49.37%	90.02%
LL	23,635,959	6,823,567,672	48.48%	88.86%
DD	22,735,011	6,676,165,768	49.92%	90.19%

**Table 3:** RNA-Seq sequence reads mapping to the Nile tilapia (*Oreochromis niloticus*) genome.

Samples	Total reads	Mapped reads	Uniq mapped Reads	Multiple map Reads	Reads map to “+”	Reads map to “−”
LD	89,865,174	68,241,765 (75.94%)	67,268,757 (74.86%)	973,008 (1.08%)	33,947,978 (37.78%)	34,001,692 (37.84%)
LL	47,271,918	35,538,899 (75.18%)	35,084,555 (74.22%)	454,344 (0.96%)	17,688,607 (37.42%)	17,708,979 (37.46%)
DD	45,470,022	35,330,624 (77.70%)	34,819,769 (76.58%)	510,855 (1.12%)	17,583,437 (38.67%)	17,611,225 (38.73%)

### Identification of DEGs

All DEGs between LL and LD, DD and LD, and LL and DD were determined (**Supplementary Table 1**). DESeq was used to screen DEGs with a false discovery rate (FDR) at an adjusted p-value ≤ 0.01. The results revealed that gene expression varied widely under the different photoperiod regimes. The comparison between LL and DD revealed 1,036 DEGs, with 307 up-regulated and 729 down-regulated genes (**Table 4**). When comparing LL and LD, 988 DEGs were identified, 721 of which were up-regulated, and 267 of which were down-regulated. In the comparison between DD and LD, 992 DEGs were identified, of which 498 were up-regulated and 494 were down-regulated. These DEGs were searched against the NCBI non-redundant (NR), Swiss-Prot, eggNOG, KEGG, GO, and COG databases (**Table 5**). In the two comparison groups (LD–LL and LD–DD), 14 important DEGs involved ovary development were identified (**Table 6**). These DEGs were found in KEGG pathways related to ovary development, such as the GnRH signaling pathway (ko04912), oocyte meiosis (ko04114), and progesterone-mediated oocyte maturation (ko04914) (**Supplementary Table 2**). Among the 14 DEGs, 7 were annotated genes and 7 were not annotated in the NCBI non-redundant (nr) database. Seven genes—*cacna1d*, *fshb*, *pgr*, *rec8*, *LOC100701982* (heat-shock protein HSP 90-alpha-like), *LOC100702193* (RAC-beta serine/threonine-protein kinase-like isoform 1), and *LOC100707861* (phosphatidylinositol 4,5-bisphosphate 3-kinase catalytic subunit alpha isoform-like)—were significantly upregulated in the LD–LL comparison. *LOC100706035* (adenylate cyclase type 8-like) and *LOC100710007* (adenylate cyclase type 3 isoform X1) were significantly downregulated in LD–LL. Three genes—*acna1d*, *adcy8*, and *arl2*—were significantly upregulated in LD–DD. *pde3a*, *LOC100706035* (adenylate cyclase type 8-like), *LOC100700615* (voltage-dependent L-type calcium channel subunit alpha-1F), and *LOC100711993* (insulin-like growth factor 1 receptor-like) were significantly downregulated in LD–DD. Furthermore, *cacna1d* was upregulated in LD–LL and LD–DD. *LOC100706035* (adenylate cyclase type 8-like) was downregulated in LD–LL and LD–DD. Therefore, these 14 DEGs could play an important role in investigating the molecular mechanisms of ovary development in fish under different photoperiod regimes.

**Table 4:** Number of differentially expressed genes (DEGs).

DEG set	DEG number	Up-regulated	Down-regulated
LD *vs.* LL	988	721	267
LD *vs.* DD	992	498	494
LL *vs.* DD	1,036	307	729

**Table 5:** Number of annotation DEGs.

DEG set	Total	COG	GO	KEGG	NR	Swiss-Prot	eggNOG
LD *vs.* LL	964	226	465	553	964	672	916
LD *vs.* DD	967	252	459	528	966	645	914
LL *vs.* DD	1,017	276	507	578	1,017	714	973

**Table 6:** Important DEGs involved in light-induced ovary development in the LD–LL and LD–DD comparison groups.

Number	Gene ID	Gene name	Regulation	
			LD–LL	LD–DD
1	LOC100706035	Adenylate cyclase type 8-like	Down	Down
2	LOC100710007	Adenylate cyclase type 3 isoform X1	Down	-
3	cacna1d	Voltage-dependent L-type calcium channel subunit alpha-1D-like	Up	Up
4	fshb	FSH beta subunit precursor	Up	-
5	LOC100700615	Voltage-dependent L-type calcium channel subunit alpha-1F	-	Down
6	adcy8	Adenylate cyclase type 8-like isoform X1	-	Up
7	pgr	Progesterone receptor isoform X2	Up	-
8	rec8	Meiotic recombination protein REC8 homolog	Up	-
9	LOC100711993	Insulin-like growth factor 1 receptor-like	-	Down
10	arl2	ADP-ribosylation factor-like protein 2-like isoform 1	-	Up
11	LOC100701982	Heat-shock protein HSP 90-alpha-like	Up	-
12	LOC100702193	RAC-beta serine/threonine-protein kinase-like isoform X1	Up	-
13	LOC100707861	Phosphatidylinositol 4,5-bisphosphate 3-kinase catalytic subunit alpha isoform-like	Up	-
14	pde3a	cGMP-inhibited 3″ -cyclic phosphodiesterase A-like isoform X2	-	Down

### Gene Functional Annotation

DEGs were identified under different conditions. GO is an international standardized gene functional classification system which can be used to assign properties to genes and their products in any organism. GO and KEGG pathway analyses were performed to understand the biological functions of the DEGs and to identify genes potentially involved in light regulation of potato tuber formation. Mapping each DEG to the terms of the GO database enabled annotation. All unigenes and DEGs could be grouped into the cellular component, molecular function, and biological process categories, respectively, in the LD–LL, LD–DD, and LL–DD (**Supplementary Figures 1**–**3**). The main biological processes and cellular processes were metabolic, as well as the responses to environmental stimulation.

**Figure 1:**
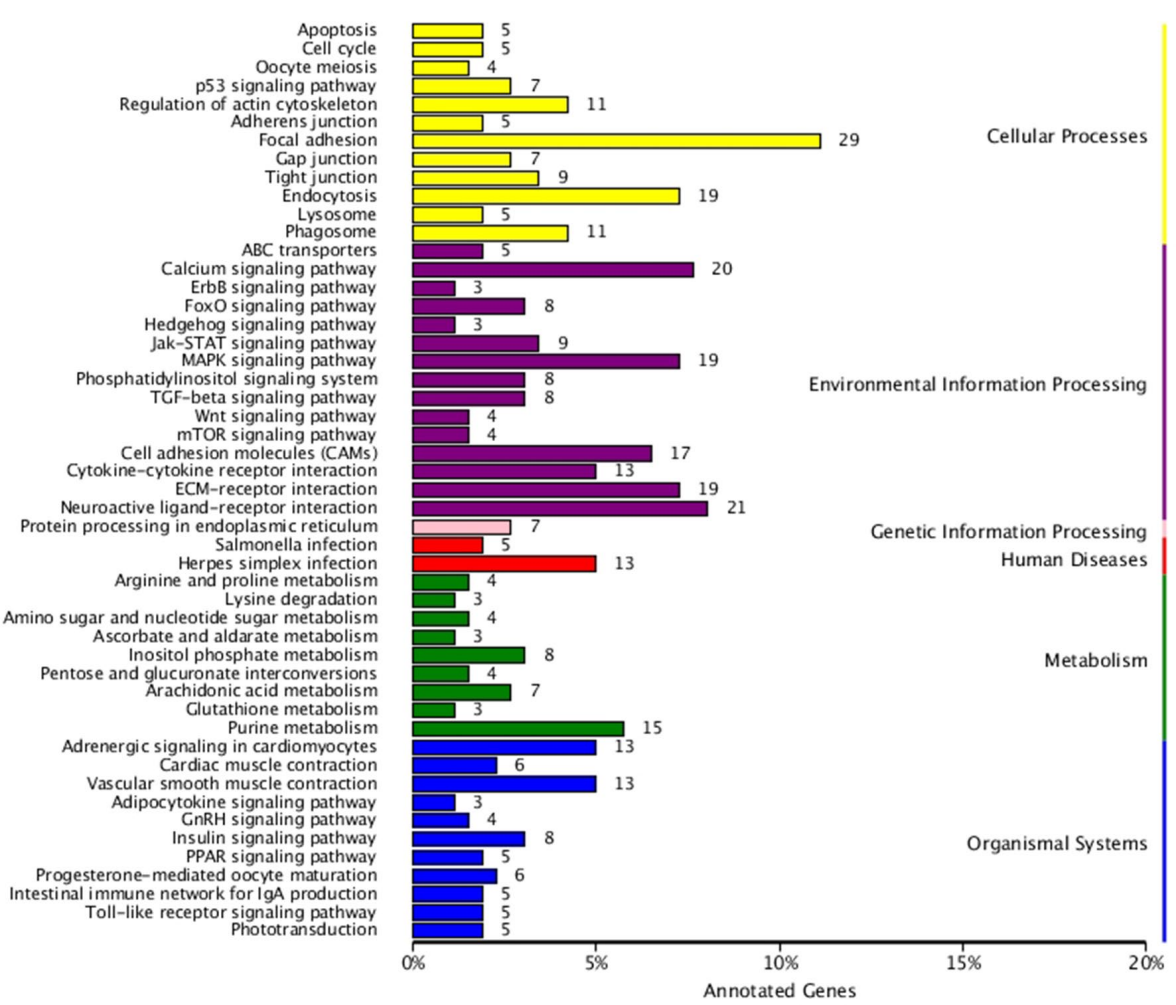
KEGG classification of differentially expressed genes (DEGs) in LD *vs.* LL. The ordinate is the name of the Kyoto Encyclopedia of Genes and Genomes (KEGG) metabolic pathway; the x-coordinate is the number of genes which are annotated to the pathway and the proportion of the total annotated genes. LD (12 h light:12 h dark), LL (24 h light:0 h dark), and DD (0 h light:24 h dark).

**Figure 2:**
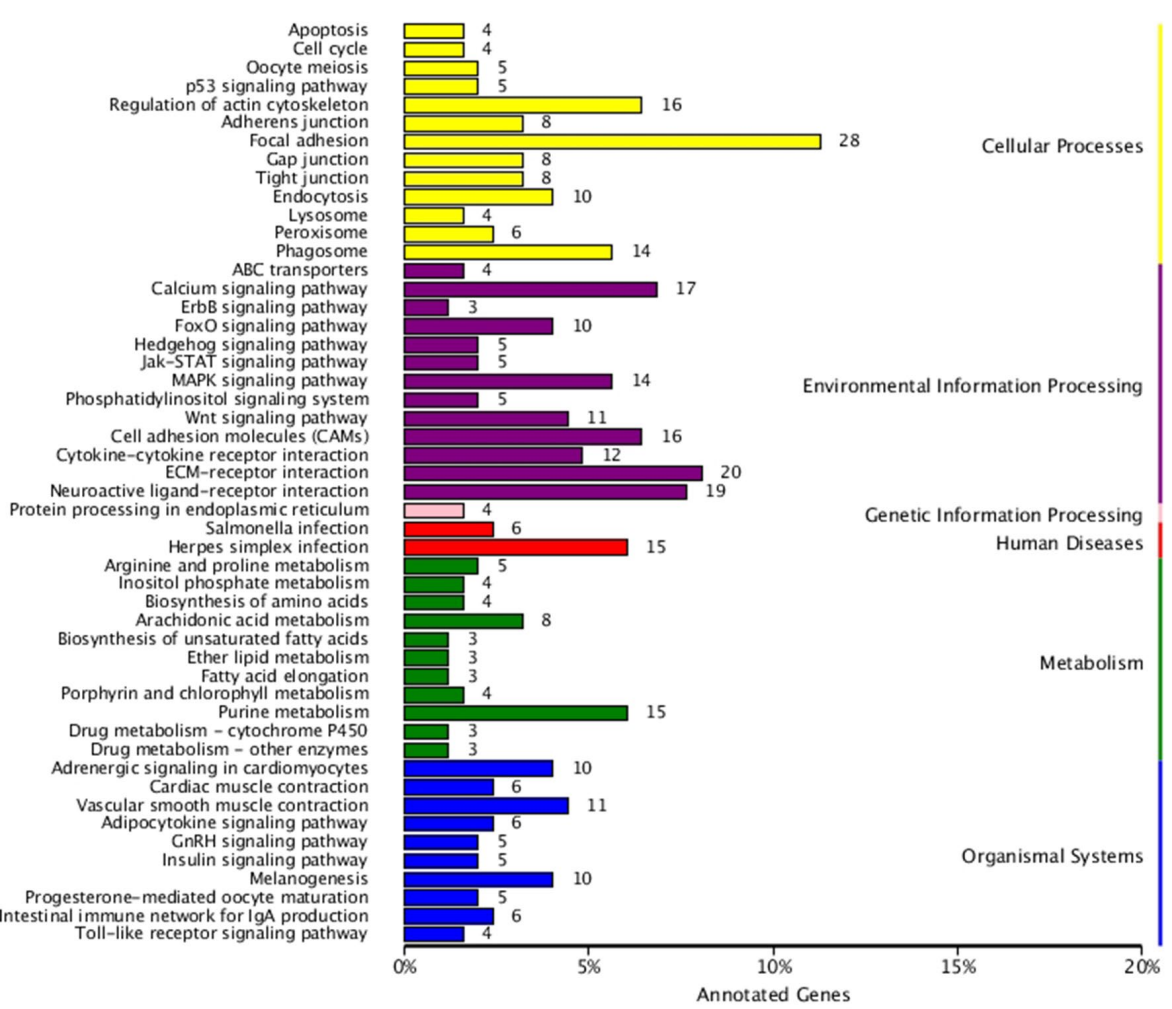
KEGG classification of DEGs in LD *vs.* DD. The ordinate is the name of the KEGG metabolic pathway; the x-coordinate is the number of genes which are annotated to the pathway and the proportion of the total annotated genes. LD (12 h light:12 h dark), LL (24 h light:0 h dark) and DD (0 h light:24 h dark).

**Figure 3:**
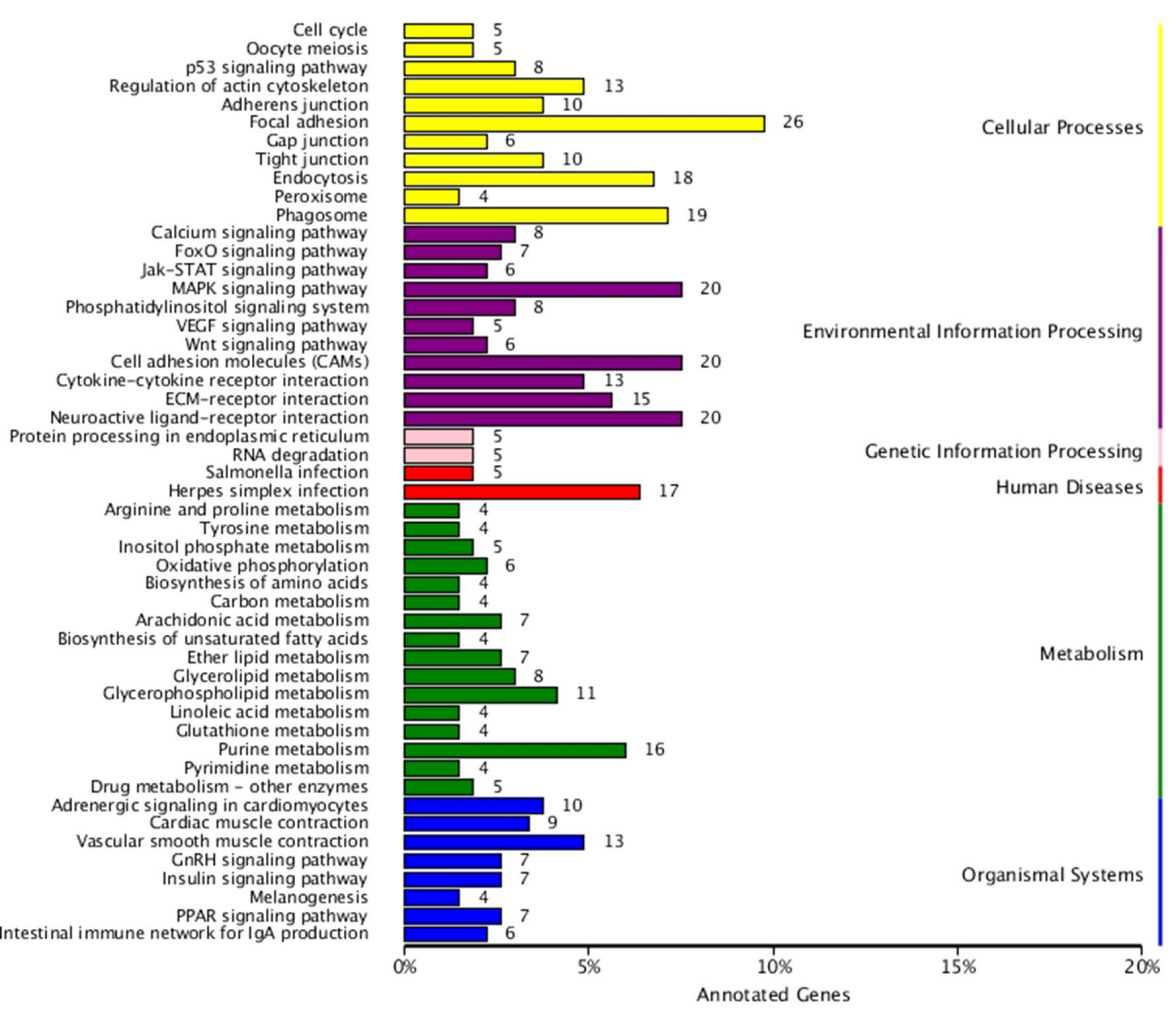
KEGG classification of DEGs in LL *vs.* DD. The ordinate is the name of the KEGG metabolic pathway; the x-coordinate is the number of genes which are annotated to the pathway and the proportion of the total annotated genes. LD (12 h light:12 h dark), LL (24 h light:0 h dark), and DD (0 h light:24 h dark).

KEGG enrichment analysis was also performed for DEGs identified in the three comparisons according to the KEGG database. Pathways with a p-value ≤ 0.05 were significantly enriched. A total of 108, 114, and 121 KEGG pathways were differentially expressed in the LD–LL, LD–DD, and LL–DD comparisons, respectively (**Figures 1**–**3**). Many significantly enriched KEGG pathways are involved in sex determination and ovarian development, such as focal adhesion, the MAPK signaling pathway, calcium signaling pathway, GnRH signaling pathway, ECM-receptor interaction, adrenergic signaling in cardiomyocytes, oocyte meiosis, progesterone-mediated oocyte maturation, and neuroactive ligand-receptor interactions, which were identified in the LD–LL and LD–DD comparisons. In the LL–DD comparison, many significantly enriched KEGG pathways were from the environmental information processing and organismal system categories. KEGG pathways involved in ovarian development were the GnRH signaling pathway, oocyte meiosis, and progesterone-mediated oocyte maturation.

### Ovary Histological Analysis

**Figure 4:**
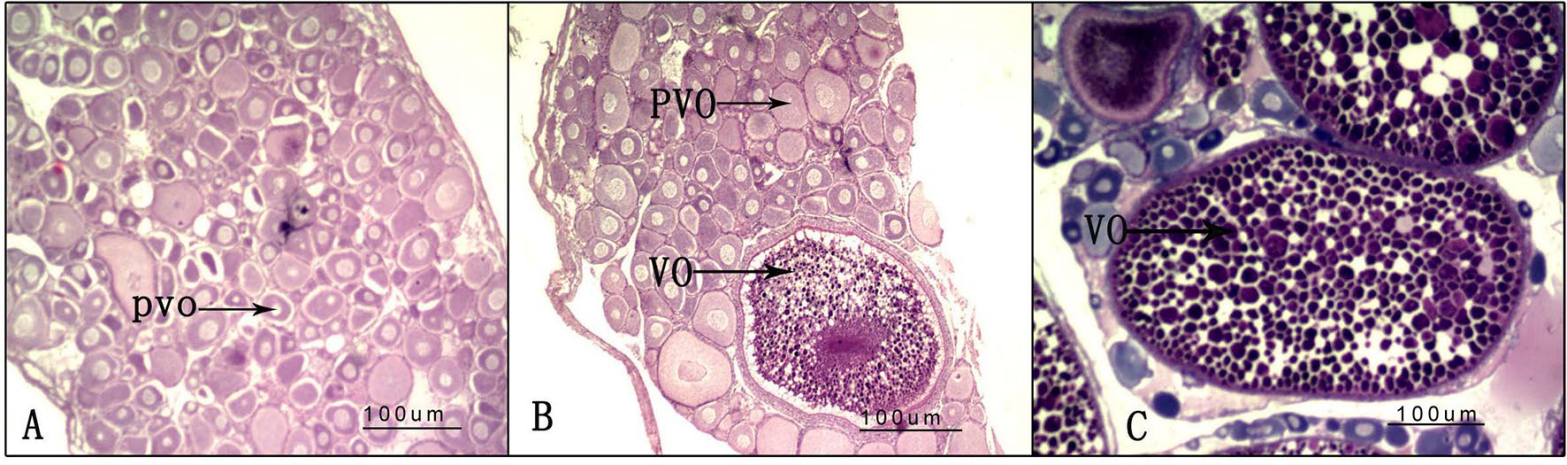
Ovarian histology under different photoperiod treatments. **(A)** 0-h light:24-h dark photoperiod; **(B)** 12-h light:12-h dark photoperiod; **(C)** 24-h light:0-h dark photoperiod. PVO, oocytes at the pre-vitellogenic stage; VO, oocytes at the vitellogenic stage.

After the experiment, nine randomly selected ovaries from the three groups (LD, LL, and DD) were used for histological analysis; one histological slice is presented for each group in **Figure 4**. In the DD group, all of the oocytes were at the pre-vitellogenic stage. There were many oocytes at the pre-vitellogenic stage and a small number of oocytes were at the vitellogenic stage in the ovaries of the LD group compared with the DD group. However, there were many oocytes at the pre-vitellogenic stage and a small number of oocytes at the vitellogenic stage in the ovaries of the LL group compared with the LD group.

### Reference Gene qRT-PCR Validation

**Figure 5:**
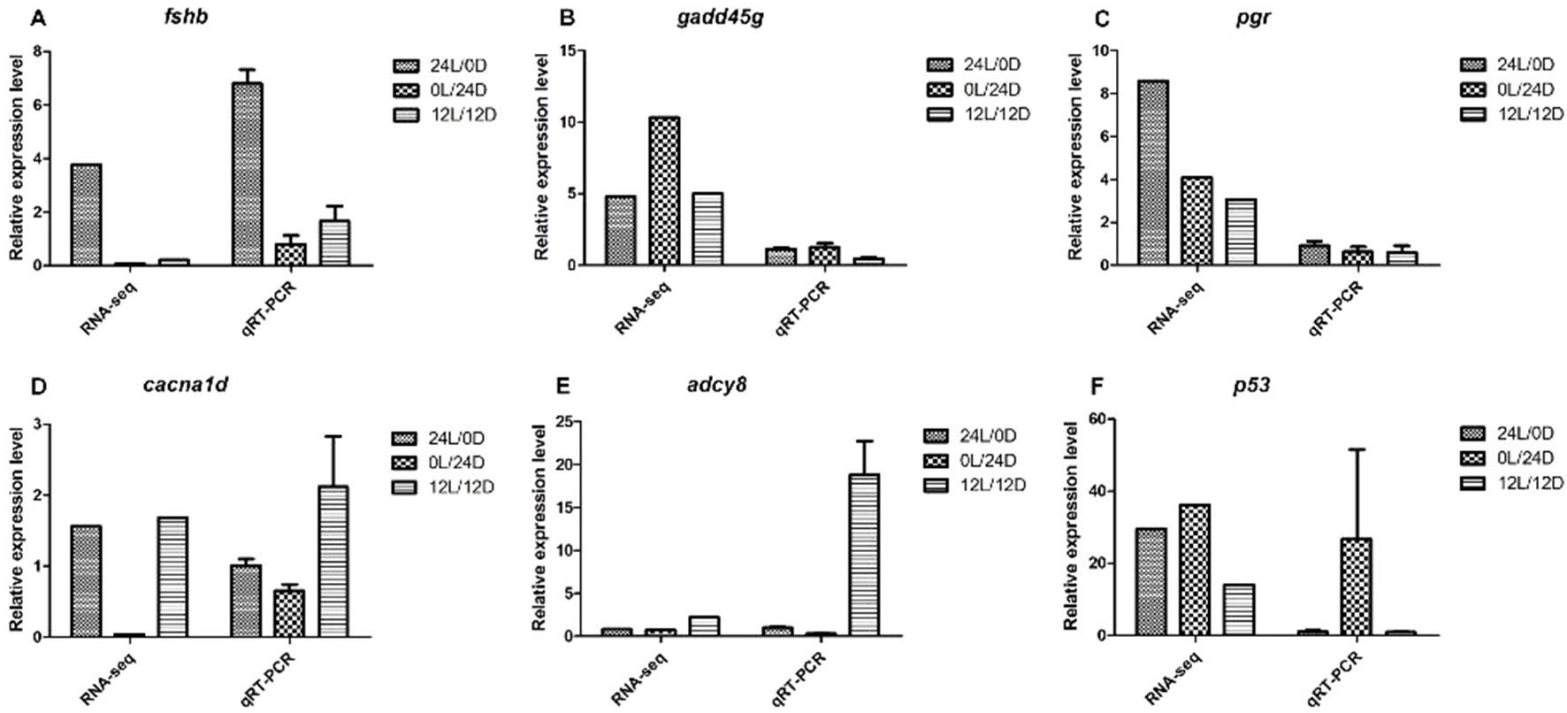
qRT-PCR validation of RNA-Seq data. **(A)***fshb*; **(B)***gaddg45*; **(C)***pgr*; **(D)***cacna1d*; **(E)***adcy8*; **(F)***p53*. The left Y-axis represents the relative expression level determined by qRT-PCR, and the right Y-axis represents FPKM determined by RNA-Seq. The scales of the left and right Y-axis are different. All data represent the mean value of three biological replicates. Error bars represent the standard errors of three replicates.

To validate the accuracy of the RNA-Seq data, a total of six DEGs with signiﬁcantly different expression levels were subjected to qRT-PCR analysis. These identified genes were: *fshb*, *gaddg45*, *pgr*, *cacna1d*, *adcy8*, and *p53*. Among them, five DEGs involved in ovary development—*fshb*, *pgr*, *cacna1d*, *adcy8*, and *gaddg45* (growth arrest and DNA damage-inducible protein GADD45 gamma-like)—were involved in growth arrest and DNA damage. The gene *p53* (cellular tumor antigen p53-like) was involved in growth arrest, DNA repair, and apoptosis. These total RNA samples were taken from nine ovaries of Nile tilapia, including the LD, LL, and DD groups. Genes displayed similar expression patterns both in RNA-Seq and qRT-PCR analyses (**Figure 5**). The consistent expression tendencies confirmed that RNA-Seq accurately quantified ovarian gene expression in the LD, LL, and DD groups.

## Discussion

The manipulation of photoperiods serves as an effective method for manipulating somatic growth and ovarian development in a variety of fish species (Campos-Mendoza et al., 2004). Relevant to this present study, artificial lighting has been shown to be influential on gonadal development in Nile tilapia during the fingerling stage (Navarro et al., 2015). In this study, gonad histological slices revealed observable differences in the LL, LD, and DD groups. Ovaries developed earlier under the longer photoperiod treatment (LL group) than in the natural light photoperiod treatment (DD group) or in darkness (LD group). Previous studies demonstrated that reproductive performance and seed production in tilapia species (*O. niloticus* and *Oreochromis spilurus*) are influenced by photoperiod manipulation (Rad et al., 2006). The experimental results presented here are consistent with these reports.

The molecular mechanism responsible for light-induced ovary development in Nile tilapia is still largely unknown. However, high-throughput sequencing of ovarian development in Pacific abalone (*Haliotis discus hannai*) and *Xenopus laevis* under various photoperiod conditions has been reported (Yu et al., 2018). The technique of RNA-Seq is both accurate and sensitive, detecting transcripts even with low abundance, such as those encoding transcription factors. In this study, the ovaries of Nile tilapia were used for transcriptome analyses, and a series of different photoperiod treatment-related genes were identified. This study identified 988, 992, and 1,036 DEGs in the LD–LL, LD–DD, and LL–DD comparisons, respectively. This indicates that, under the different photoperiod regimes, changes in the expression of many genes were noted. DEGs associated with ovarian development in a given photoperiod were identified. These genes were annotated to 108, 114, and 121 KEGG pathways. The GnRH signaling pathway, oocyte meiosis, and progesterone-mediated oocyte maturation were all involved in ovarian development in the transcriptome files of LD–LL and LD–DD. From the DEGs in these three pathways, 14 important DEGs involved in light-induced ovary development were identified in the two comparison groups (LD–LL and LD–DD). Seven genes whose expression levels changed after long photoperiod treatments (LD–LL comparison) were upregulated. These included *cacna1d*, *fshb*, *pgr*, *rec8*, *LOC100701982* (heat-shock protein HSP 90-alpha-like), *LOC100702193* (RAC-beta serine/threonine-protein kinase-like isoform X1), and *LOC100707861* (phosphatidylinositol 4,5-bisphosphate 3-kinase catalytic subunit alpha isoform-like). Two genes whose expression levels changed after long photoperiod treatments (LD–LL comparison) were downregulated, such as *LOC100706035* (adenylate cyclase type 8-like) and *LOC100710007* (adenylate cyclase type 3 isoform X1). Expression levels of variation of the above genes indicated their potential functions in light-induced ovarian development.

*Fshb* plays an important role in regulating gonadal activities such as gametogenesis, steroidogenesis, and maturation (Swanson et al., 2003). In the present study, the expression of *fshb* was upregulated in the LD–LL comparison, with no change was found in the LD–LL comparison. Furthermore, the relative expression level of *Fshb* from transcriptome analysis was confirmed with qRT-PCR measurements (**Figure 5**). From these findings, we conclude that *Fshb* may be an important candidate gene for ovarian development.

Fish reproduction is generally regulated by the interaction of endogenous neuroendocrine, endocrine, and autocrine/paracrine signals with environmental factors (Baroiller and Guiguen, 2001; Segner et al., 2006). These factors include food availability, temperature, and season. In this study, the data indicated that several KEGG pathways are involved in environmental information processing and ovarian development, such as the MAPK signaling pathway, neuroactive ligand-receptor interaction, GnRH signaling pathway, oocyte meiosis, and progesterone-mediated oocyte maturation (**Supplementary Table 2**). Fish reproductive cycles are controlled by an intricate interplay between the hypothalamus, pituitary, and gonads. Gonadotropin-releasing hormone (GnRH) is the main hypothalamic regulator of the reproductive system in teleost fish (Gur et al., 2002). GnRH regulates the synthesis and release of gonadotropin, luteinizing hormone (LH), and follicle-stimulating hormone (FSH). Mitogen-activated protein kinase (MAPK) activities represent regulation by GnRH that is necessary for normal fertility (Bliss et al., 2010). Among these ovary-related genes, several ovarian development-related genes are differentially expressed in ovarian tissues. Therefore, we focused on the GnRH signaling pathway in this study. There were some DEGs in the KEGG pathway of the GnRH signaling pathway, such as *LOC100700615* (voltage-dependent L-type calcium channel subunit alpha-1F), *LOC100703685* (transcription factor AP-1-like), *adcy8*, *fshb*, *jun*, *mmp2*, and *pla2g4a* in LD–LL comparison groups (**Supplementary Table 2**). The FSH is involved in the initiation of gametogenesis and regulation of gonadal growth (Mateos et al., 2002). Follicle-stimulating hormone (Fsh) synthesized in the pituitary gland is a glycoprotein hormone consisting of a common a-subunit and a hormone-specific b-subunit (Horie et al., 2014). In this study, the expression level of *fshb* was upregulated in the LD–LL comparisons.

Both RNA-Seq and bioinformatics tools were used to reevaluate transcriptome files of ovarian development under different photoperiod regimes. These analyses revealed the molecular mechanisms involved in light-induced ovarian development. Application of these approaches will expand our current knowledge of the molecular mechanisms involved in light-induced ovarian development in Nile tilapia. The present results have provided the basis and reference for fish reproductive physiology.

## Data Availability

All RNA-Seq data generated in this study have been deposited in the NCBI SRA database (SRA accession: SRP150481).

## Ethics Statement

All experiments were performed according to the Guide for the Care and Use of Laboratory Animals of China. This study was approved by the Committee on the Ethics of Animal Experiments of Guangxi Academy of Fishery Sciences.

## Author Contributions

LZ and YL conceived and designed the experiments; ZT and YZ performed the experiments; JX, HZ, WM, ZG, and XZ analyzed the data; ZT wrote the paper; all authors have read and approved the final manuscript.

## Funding

This research was supported by the Guangxi Science and Technology Research Program (AA17204094-2), the Natural Science Foundation of China (31560716, 31672627, 31760756), the Guangxi Natural Science Foundation (2015GXNSFBA139108, 2016GXNSFFA380002), Guangxi Hundred-Talent Program, Training Project of High-level Professional and Technical Talents of Guangxi University.

## Conflict of Interest Statement

The authors declare that the research was conducted in the absence of any commercial or financial relationships that could be construed as a potential conflict of interest.

## Supplementary Material

Supplementary Table 1 Detailed information of the differentially expressed genes in the three groups.

Supplementary Table 2 KEGG Path in 3 comparison groups

Supplementary Figure 1 The GO annotation classification chart of differentially expressed genes in LD vs LL groups. Histogram presentation of Gene Ontology (GO) classification. The results are summarized in three main categories: biological process, cellular component, and molecular function. The right y-axis indicates the number of genes in a category. The left y-axis indicates the percentage of a specific category of genes in that main category. LD (12 h light: 12 h dark), LL (24 h light: 0 h dark) and DD (0 h light: 24 h dark).

Supplementary Figure 2 The GO annotation classification chart of differential expression genes in LD vs DD. Histogram presentation of Gene Ontology classification (GO). The results are summarized in three main categories: biological process, cellular component, and molecular function. The right y-axis indicates the number of genes in a category. The left y-axis indicates the percentage of a specific category of genes in that main category. LD (12 h light: 12 h dark), LL (24 h light: 0 h dark) and DD (0 h light: 24 h dark).

Supplementary Figure 3 The GO annotation classification chart of differential expression genes in LL vs DD. Histogram presentation of Gene Ontology classification (GO). The results are summarized in three main categories: biological process, cellular component, and molecular function. The right y-axis indicates the number of genes in a category. The left y-axis indicates the percentage of a specific category of genes in that main category. LD (12 h light: 12 h dark), LL (24 h light: 0 h dark) and DD (0 h light: 24 h dark).
