# Supplementary figures and images for: Transcriptome Analysis of Ovary Development in Nile Tilapia Under Different Photoperiod Regimes
Source: Front Genet. 2019 Sep 19;10:894. doi: 10.3389/fgene.2019.00894 (PMC6761324; doi:10.3389/fgene.2019.00894)

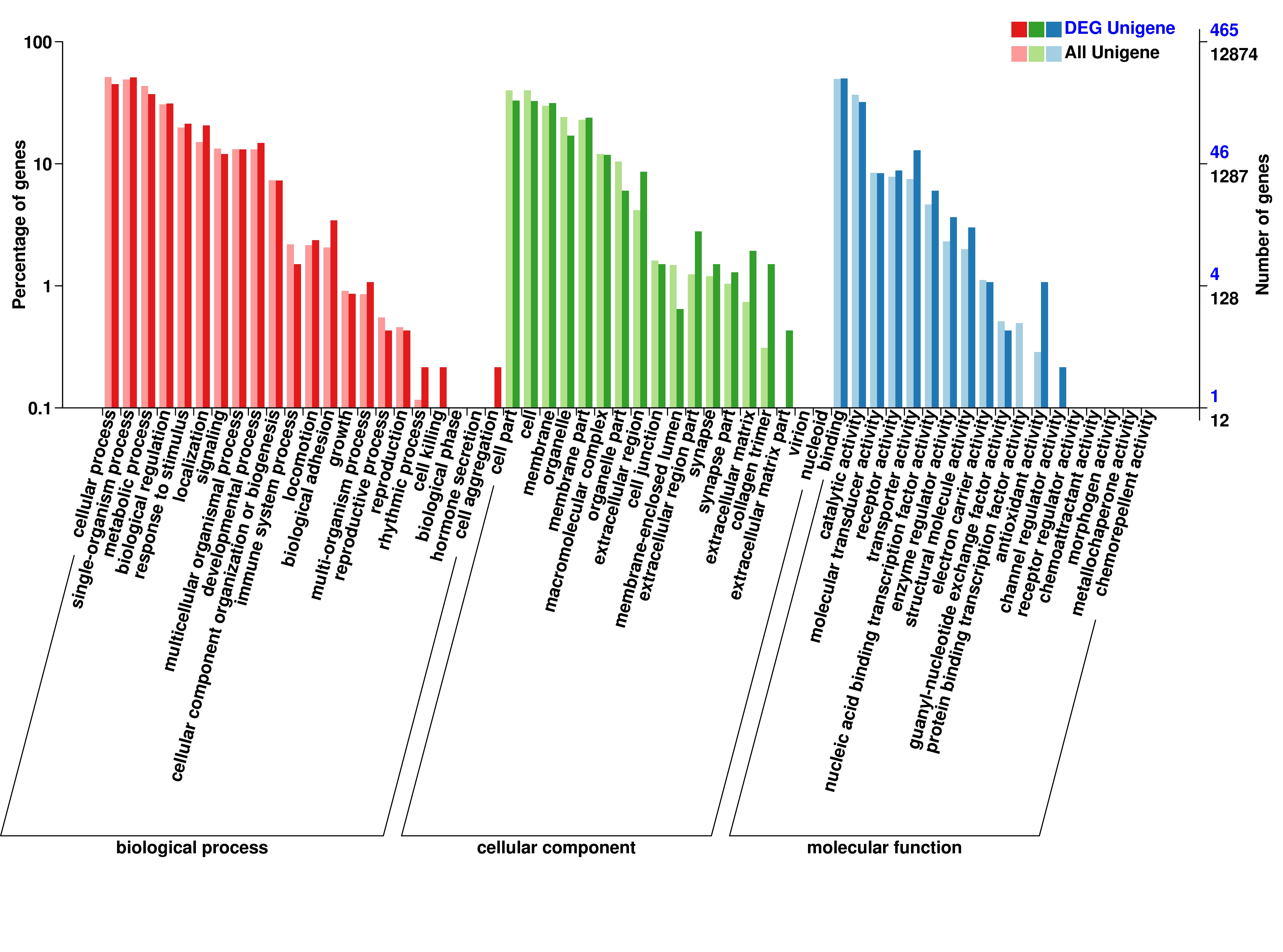

Supplement: Supplementary Figure 1 — The GO annotation classification chart of differentially expressed genes in LD vs LL groups. Histogram presentation of Gene Ontology (GO) classification. The results are summarized in three main categories: biological process, cellular component, and molecular function. The right y-axis indicates the number of genes in a category. The left y-axis indicates the percentage of a specific category of genes in that main category. LD (12 h light: 12 h dark), LL (24 h light: 0 h dark) and DD (0 h light: 24 h dark). [file Image_1.jpeg]

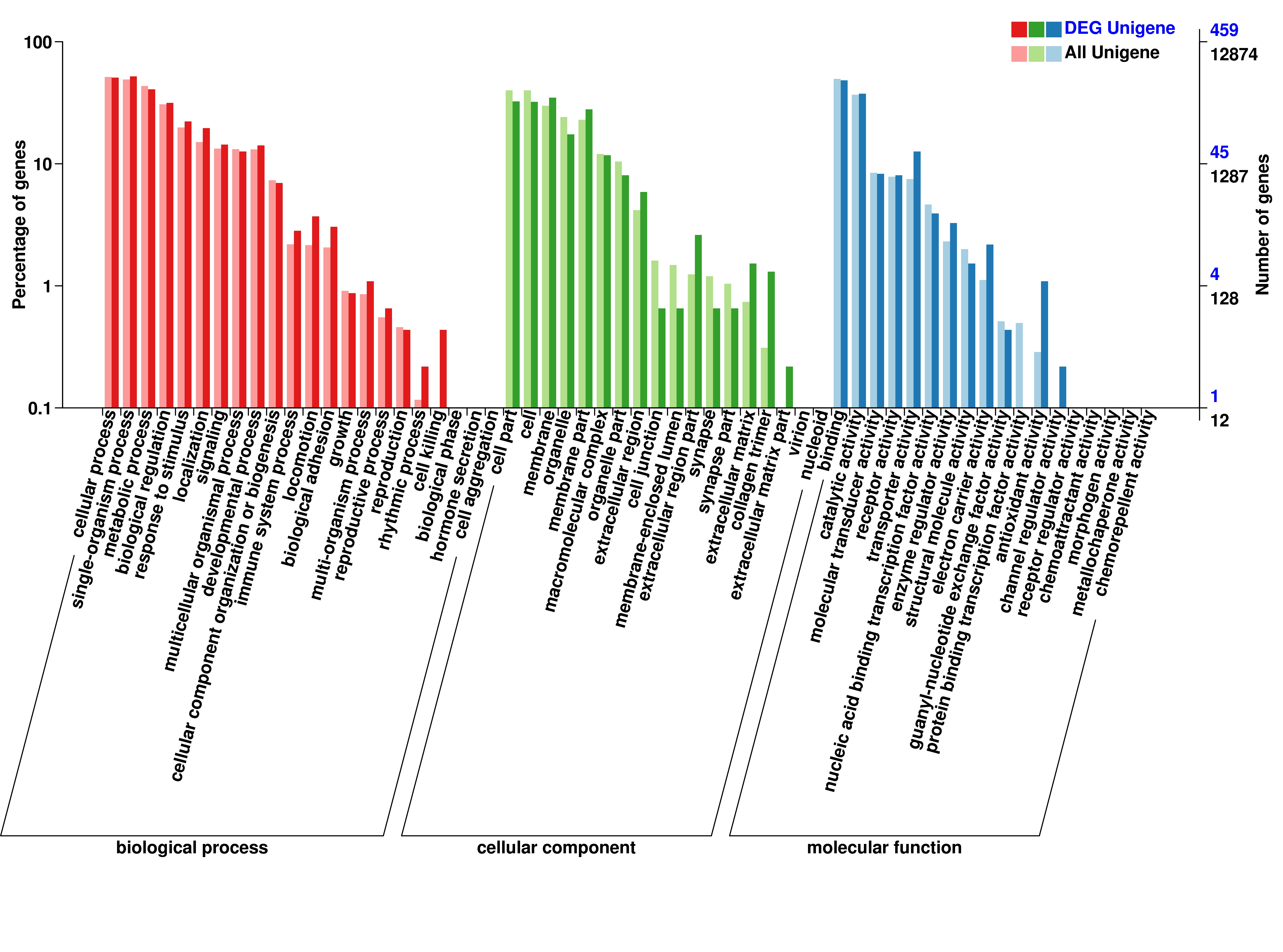

Supplement: Supplementary Figure 2 — The GO annotation classification chart of differential expression genes in LD vs DD. Histogram presentation of Gene Ontology classification (GO). The results are summarized in three main categories: biological process, cellular component, and molecular function. The right y-axis indicates the number of genes in a category. The left y-axis indicates the percentage of a specific category of genes in that main category. LD (12 h light: 12 h dark), LL (24 h light: 0 h dark) and DD (0 h light: 24 h dark). [file Image_2.jpeg]

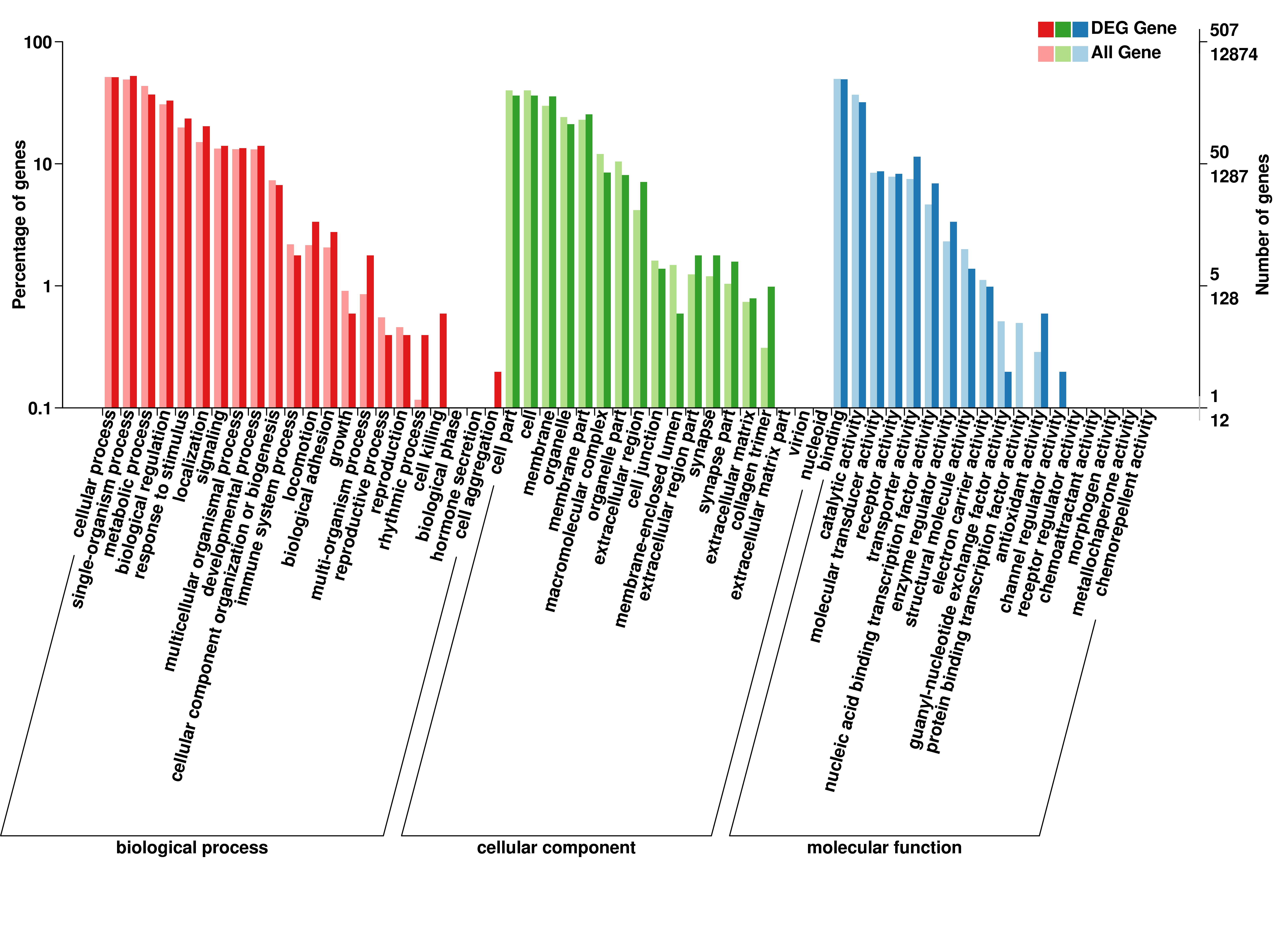

Supplement: Supplementary Figure 3 — The GO annotation classification chart of differential expression genes in LL vs DD. Histogram presentation of Gene Ontology classification (GO). The results are summarized in three main categories: biological process, cellular component, and molecular function. The right y-axis indicates the number of genes in a category. The left y-axis indicates the percentage of a specific category of genes in that main category. LD (12 h light: 12 h dark), LL (24 h light: 0 h dark) and DD (0 h light: 24 h dark). [file Image_3.jpeg]
